# No Medium-Term Spinocerebellar Input Plasticity in Deep Cerebellar Nuclear Neurons In Vivo?

**DOI:** 10.1007/s12311-016-0839-0

**Published:** 2016-12-28

**Authors:** Hannes Mogensen, Fredrik Bengtsson, Henrik Jörntell

**Affiliations:** Neural Basis of Sensorimotor Control, Department of Experimental Medical Science, Lund University, BMC F10, Tornavägen 10, 221 84 Lund, Sweden

**Keywords:** Plasticity, Deep cerebellar nuclear neurons, Mossy fibers, Climbing fibers

## Abstract

The existence of input plasticity in the deep cerebellar nuclear (DCN) cells of the adult cerebellum could have profound implications for our understanding of cerebellar function. Whereas the existence of plastic changes in mossy fiber (mf) synaptic responses in DCN neurons has been demonstrated in juvenile slices, there has so far been no direct demonstration of this form of plasticity in the adult cerebellum in vivo. In the present paper, we recorded from neurons in the anterior interposed nucleus (AIN) and stimulated the spinocerebellar tracts (SCT) directly or via the skin to obtain mf activation and the inferior olive to activate climbing fibers (cfs) in the nonanesthetized, adult, decerebrated cat. We used three different types of protocols that theoretically could be expected to induce plasticity, each of which involved episodically intense afferent activation lasting for 10 min. These were conjunctive mf-cf activation, which effectively induces plasticity in cortical neurons; mf and cf activation in a pattern resembling the protocol for inducing classical conditioning; and conjunctive activation of two excitatory mf inputs. None of these protocols had any statistically significant effect on the evoked responses in the AIN neurons. We conclude that the input plasticity for excitatory mfs in the AIN cells of the adult cerebellum in vivo is likely to be less effective than that of parallel fiber synaptic inputs in cerebellar cortical cells, at least in the timespan of 1 h.

## Introduction

Plasticity in the neurons of the deep cerebellar nuclei as a substrate for behavioral learning has been debated for a long time, and theoretical predictions and circumstantial evidence have been used to argue that it may be required in some situations of presumed cerebellar-dependent adaptation [7, 24, 25, 29, 34]. Plasticity of deep cerebellar nuclear (DCN) neuron intrinsic excitability [1] and mossy fiber (mf) inputs [26, 27] have been demonstrated in vitro, but in these cases, only for slice preparations of the juvenile cerebellum. Whereas major changes in the circuitry structure and physiology may be expected to occur during development, cerebellar adaptation works also in adult life and it is important for our understanding of the functioning of the cerebellum to know whether these changes can occur and consequently contribute to adaptation and learning also during adult life. A recent indication that this may be the case was a study where sprouting of mf axons in the DCN was observed after a period of intense training over several days, but this is typically one order of magnitude slower than the timescale on which the cerebellar adaption is believed to work [5]. In any case, it remains to be shown that the effective input to the DCN neurons increases under these conditions.

From a limb control point of view, an important source of information to the neurons of the interpositus nuclei comes from the spinocerebellar and spinoreticulocerebellar systems (SCTs). These are among the few mf systems that have been shown to directly innervate DCN neurons through collaterals of their axons that pass by the nuclei before they form mf synapses in the cortex [3, 19–23, 33]. These mf systems sample information about ongoing activity in spinal sensorimotor circuits, which may be crucial for our capacity to achieve limb intersegment coordination [31]. Hence, synaptic plasticity in the mf-DCN connections of these systems could theoretically alter the conditions for limb coordination control. A consistent relationship between the location of the cutaneous climbing fiber (cf) receptive field and the distribution of skin areas from which excitatory inputs were evoked in anterior interposed nucleus (AIN) neurons was recently described [3]. This relationship suggests the presence of a cf-dependent plasticity mechanism for regulating the excitatory mf inputs to DCN neurons. Alternatively, the location of the cf receptive field reflects the motor control function of the DCN cell [6, 13], and the synapses of the spinocerebellar tract (SCT) mfs most frequently associated with that specific motor control function could be strengthened through activity-dependent mechanisms, possibly NMDA-receptor dependent [27], triggered by the degree of correlated pre- and postsynaptic activity.

**Fig. 1:**
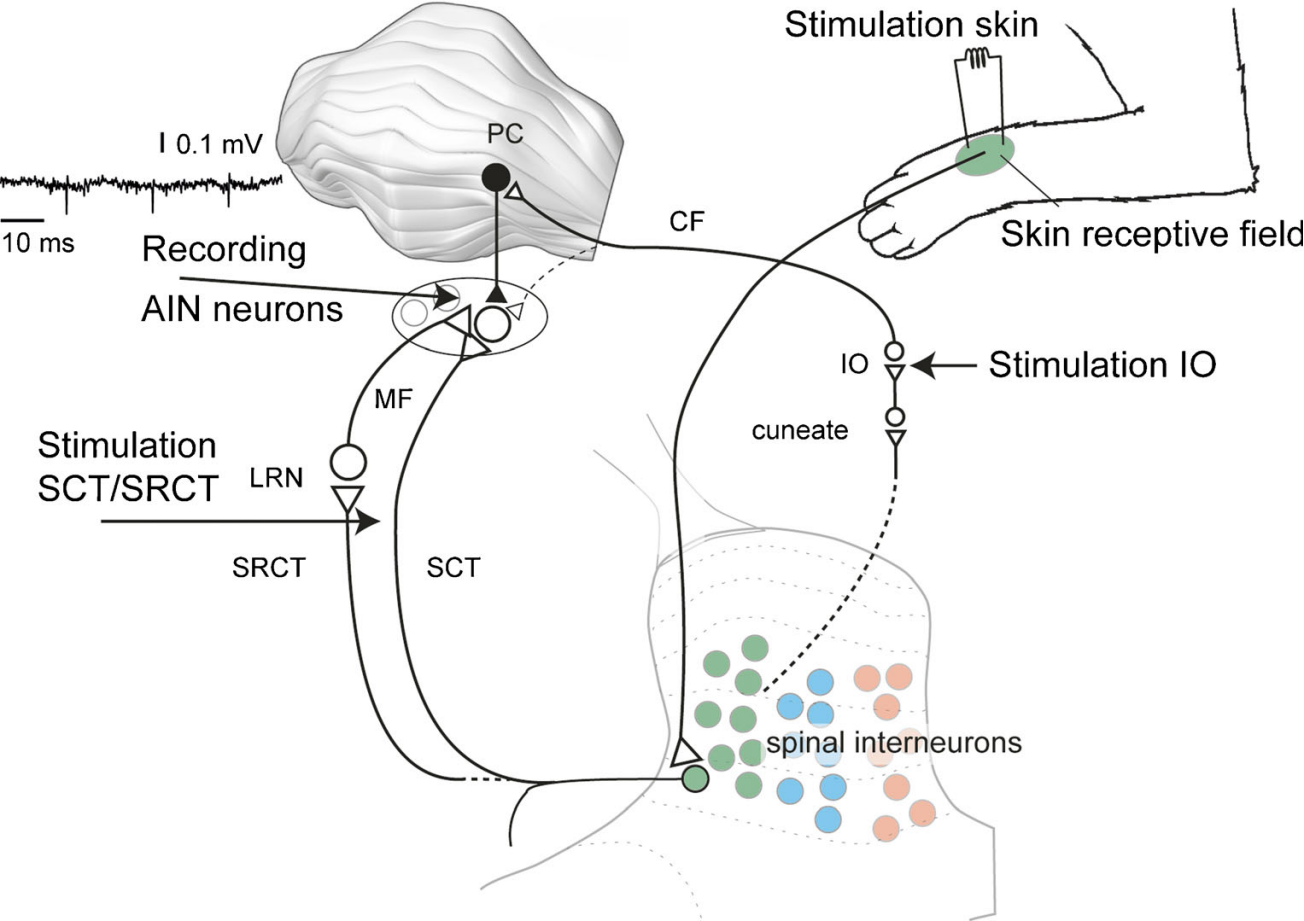
Targeted circuitry structures. Recordings were made from neurons of the anterior interposed nucleus (*AIN*). Direct electrical activation at the level of the lateral reticular nucleus (*LRN*) in the brainstem presumably activated both fibers of passage of the spinocerebellar tracts (*SCTs*) and the spinoreticulocerebellar tract or pathway (*SRCT*), where the latter represents spinal interneuron information that is forwarded to the cerebellum after a synapse in the LRN. SCT and SRCT pathways, which make mossy fiber (*MF*) synapses with the AIN neurons, were also activated using electrical stimulation of the skin. Importantly, the electrical skin stimulation was made from specific receptive fields that did not overlap the climbing fiber (*CF*) receptive field of the Purkinje cells (*PCs*) that were afferent to the AIN neuron recorded from [4, 6]. Climbing fiber activation was achieved by direct electrical stimulation in the inferior olive (*IO*).

In the present study, we addressed the issue of mf-DCN neuron plasticity using direct electrical activation of the SCTs or skin stimulation to activate the cutaneously activated components of the SCTs. These inputs were combined with each other to address the possible induction mechanism described above. To also explore protocols known to be effective at the cortical level, we combined the SCT input with conjunctive cf input [14] or, alternatively, let the train of SCT stimulation be followed by cf activation, i.e., a similar temporal relationship as in classical conditioning protocols [11] (Fig. 1). The inputs were repeated at high intensity for 10 min, and the effects on the mf-AIN input were tracked for about 1 h. We find that neither of the three protocols produces any statistically significant change in the AIN cell responses to inputs from the mf pathways. The contrast with the previously described dramatic input plasticity effects in the neurons of the cerebellar cortex, using similar protocols, is discussed.

## Materials and Methods

### Ethics Statement

The experimental procedures were approved in advance by the Malmö/Lund Animal Research Ethics Committee (permit number and approval-ID: M32-09 and M05-12). Initial surgery was performed under propofol anesthesia, and all efforts were made to minimize suffering. Our EEG recordings were characterized by a background of periodic 1–4 Hz oscillatory activity, periodically interrupted by large-amplitude 7–14 Hz spindle oscillations lasting for 0.5 s or more. These forms of EEG activities are normally associated with deep stages of sleep. The pattern of EEG activity and the blood pressure remained stable and did not change with noxious stimulation throughout experiments.

### Preparation

Adult cats (*N* = 14) were prepared as previously described. Briefly, following an initial anesthesia with propofol (Diprivan® Zeneca Ltd., Macclesfield Cheshire, UK), the animals were decerebrated at the intercollicular level and the anesthesia was discontinued. The animals were artificially ventilated and the end-expiratory CO_2_, blood pressure, and rectal temperature were continuously monitored and maintained within physiological limits. Mounting in a stereotaxic frame, drainage of cerebrospinal fluid, pneumothorax, and clamping the spinal processes of a few cervical and lumbar vertebral bodies served to increase the mechanical stability of the preparation. The dorsal part of the pars intermedia of the left cerebellum was exposed to allow microelectrode access to the AIN. An additional exposure was made of the brainstem/spinal cord junction between the base of the skull and the first cervical vertebra. All exposed areas were covered in paraffin oil to prevent tissue drying.

### Recordings and Stimulation

Patch clamp pipettes or metal microelectrodes (tungsten-in-glass microelectrodes, exposed tip 10–20 μm) were advanced to target the AIN as previously described [3, 4]. All neurons included in this study were putative glutamatergic projections neurons, based on the preponderance of short (<25 ms) interspike intervals and intermediate spike-widths [4]. We recorded neurons from both forelimb and hindlimb regions of this nucleus, as identified using the location of the cf receptive field of the afferent Purkinje cells (Fig. 1). This location can be mapped out using electrical stimulation of the skin (0.1 ms pulses of 1.0 mA applied through percutaneous needle electrodes [16])—if the cfs of the locally afferent Purkinje cells are activated by the stimulation, characteristic local field potentials [6, 8] and postinhibitory rebound responses of the DCN neurons can be recorded [4]. In this way, the location of the cf receptive field can be identified.

In order to stimulate the spinocerebellar and spinoreticulocerebellar tracts, which provide direct mf synaptic inputs to the interpositus nuclei, we placed a tungsten-in-glass microelectrode (exposed tip 50–150 μm) for stimulation laterally at the border between the spinal cord and brainstem. Using this stimulation microelectrode, mf field potentials recorded inside the AIN were routinely evoked at threshold intensities of <20 μA (single stimulus pulse of 0.1 ms duration), suggesting an effective recruitment of directly and synaptically activated (via the lateral reticular nucleus) mf synapses. In addition, we used electrical skin stimulation (pair of percutaneous needle electrodes with 5–10 mm spacing, stimulated at 1 mA shocks with 0.1 ms duration) to recruit another putative pool of spinocerebellar mfs. Cutaneous input is known to activate parts of the spinocerebellar neuron population, and since the other pathway mediating cutaneously activated mf input, the main cuneate nucleus does not terminate in the AIN [9]; potent excitatory responses evoked from the skin [3] are likely due to spinocerebellar mfs which should be at least partly non-overlapping with the population of mfs activated from the brain stem. The skin stimulation used was verified to not activate the afferent cfs and evoked a monophasic excitatory response [3].

In order to activate cfs, a second stimulation electrode was placed in the inferior olive, where low-threshold cf responses (evoked at <10 μA) could be evoked in the pars intermedia of the cerebellar cortex and in the AIN [4, 15].

### Protocols

Using the SCT or skin stimulation as test stimulation, we applied three different stimulation protocols to investigate whether plasticity in the input to the AIN neurons could be recorded. In most cases, more than one protocol was applied in the same experiment. When this was the case, the recording electrode was moved to a different location in the AIN, where neurons had substantially different location of their cf receptive fields (i.e., hindlimb versus forelimb, or proximal versus distal parts of the limb). We also moved the SCT stimulation electrode to recruit a different set of mfs, and also the skin stimulation used to evoke mf inputs was moved to a distinctly different location. The three different protocols that we used were as follows:

1. The combined SCT and skin burst stimulation protocol. The SCT electrode was stimulated with 15 pulses at 200 Hz, and the skin was stimulated 10 times at 333 Hz. With this configuration, the two inputs evoked largely overlapping time windows of excitation. The SCT stimulation intensity was typically 30–70 μA, in a couple of cases 100 μA.
2. The skin burst and simultaneous, single inferior olive (IO) stimulation protocol. The IO was stimulated once, and a skin burst stimulation of 50 pulses at 333 Hz was started 10 ms in advance in order for the first mf input to arrive at the same time as the cf input (the mf input evoked from the periphery needs at in the order of 10 ms to reach the cerebellar nuclei [3]).
3. The skin burst and delayed single IO stimulation protocol. A skin burst stimulation of 50 pulses at 333 Hz and at the time point of the last stimulation pulse, a single-pulse IO stimulation was applied.

For all three protocols, the bursts were repeated at 0.33 Hz for 10 min, i.e., for a total of 200 repetitions.

### Analysis

We quantified the responses obtained from a single-pulse stimulation, either to the SCT or to the skin, before and after a burst stimulation protocol. For the protocols involving skin bursts and the simultaneous or delayed IO stimulation, respectively, the responses were quantified using peristimulus histograms of raw spike time data (5 ms bin width). For the display and analysis of the combined skin burst and SCT stimulation protocol, we used a kernel density estimation (KDE) plot, i.e., each spike was replaced by a Gaussian distribution with standard deviation of 0.5 ms. The averaged sum of all Gaussian distributions transforms a discrete spiking pattern into a continuous function describing the spiking probability on a continuous time scale. The standard deviation of the kernels was set so that the total spiking probability function was smooth across neurons. This was done as the responses to the SCT stimulation were brief, which reduced the total number of spikes and made the responses more sensitive to chance distributions of single spikes. The KDE helped in reducing this problem. See Hoebeek et al. [10] for a more comprehensive discussion on KDE.

In all cases, the response was quantified as the mean firing frequency during the time window of the response, with the firing frequencies being obtained either from the KDE plots or the peristimulus histograms. To smooth the signal used in the analysis, the peristimulus histograms were filtered with a moving average of width 15 ms. The response onset was counted from the first occurrence of at least two consecutive bins with an activity that exceeded the baseline activity by at least two standard deviations. The end of the response was defined as the bin where the activity decreased to the threshold. For each cell, the time window for the response was initially calculated individually for every peristimulus histogram (i.e., control and all the post-protocol time points). Then the median start and end points of the responses were used to define the response time window for the cell, in which the response was quantified. The response was quantified as the mean net activity during the defined time window. For responses evoked by SCT, the responses obtained were typically evoked between 1.5 to 4.0 ms after the onset of the stimulation. The KDE in itself did not allow a rigorous setting of the time limits, but histograms of the raw data provided a support for the chosen time limits in a similar fashion as above. For responses evoked by the skin, the quantified data was typically evoked within a response latency time window of 10–30 ms after the onset of the skin stimulation.

Subsequently, the relative response for each set of single-pulse stimulations was compared to the relative response before onset of the burst protocol and the change in response from each cell was analyzed in separate consecutive time spans of 10 min (time points). The null hypothesis that there was no net change in the response was tested using Wilcoxon signed-rank test. The signed-rank test was computed for each time point by comparing the total number of cells with the number of cells with a positive response. The probability for the outcome is calculated, assuming there is a 50% probability for each cell having a positive change in response.

## Results

**Fig. 2:**
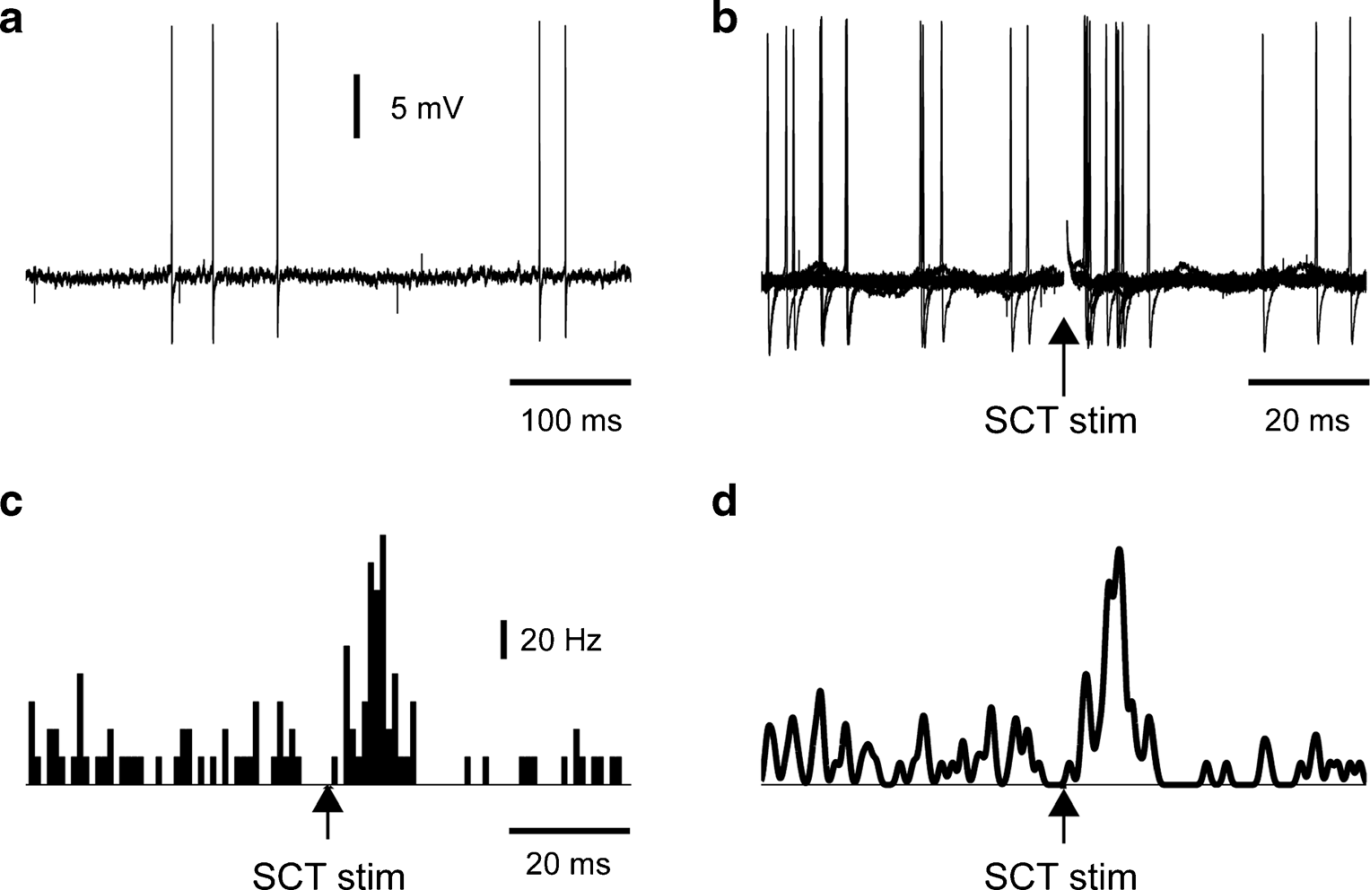
Sample AIN neuron recording and response to single shock spinocerebellar tract (*SCT*) stimulation. **a** Long raw trace of loose-patch cell-attached AIN cell recording. **b** Ten superimposed traces to illustrate the spike responses to a single shock SCT stimulation. The stimulus shock artifact was blanked for clarity. **c** Peristimulus histogram (bin width 1 ms) of responses evoked by SCT stimulation (*N* = 50 repetitions). **d** Same responses as in **c** but in this case, a kernel density estimation (KDE) curve. KDE was used to evaluate the responses for the first set of experiments as the SCT stimulation evoked fast and brief responses, for which KDE provided a better reflection

DCN neurons were recorded (Fig. 2a) in the left AIN (Fig. 1). A primary source of mf input to the AIN neurons is the spinocerebellar and spinoreticulocerebellar tracts (SCTs) (Fig. 1). Therefore, we located a stimulation electrode laterally at the level of caudal brainstem/rostral spinal cord which could stimulate the ascending SCTs on the left side (similar mediolateral location as in Bengtsson and Jorntell [3] but with a more caudal location). AIN neurons had relatively robust responses to single shock SCT stimulation (Fig. 2b), as illustrated in peristimulus histograms obtained on repeated stimulation (Fig. 2c). Figure 2d shows the relation between a peristimulus histogram and a KDE plot, which was used for the analysis of the responses evoked by SCT stimulation.

### Effects of Combined SCT Burst and Skin Burst Stimulation Protocol

**Fig. 3:**
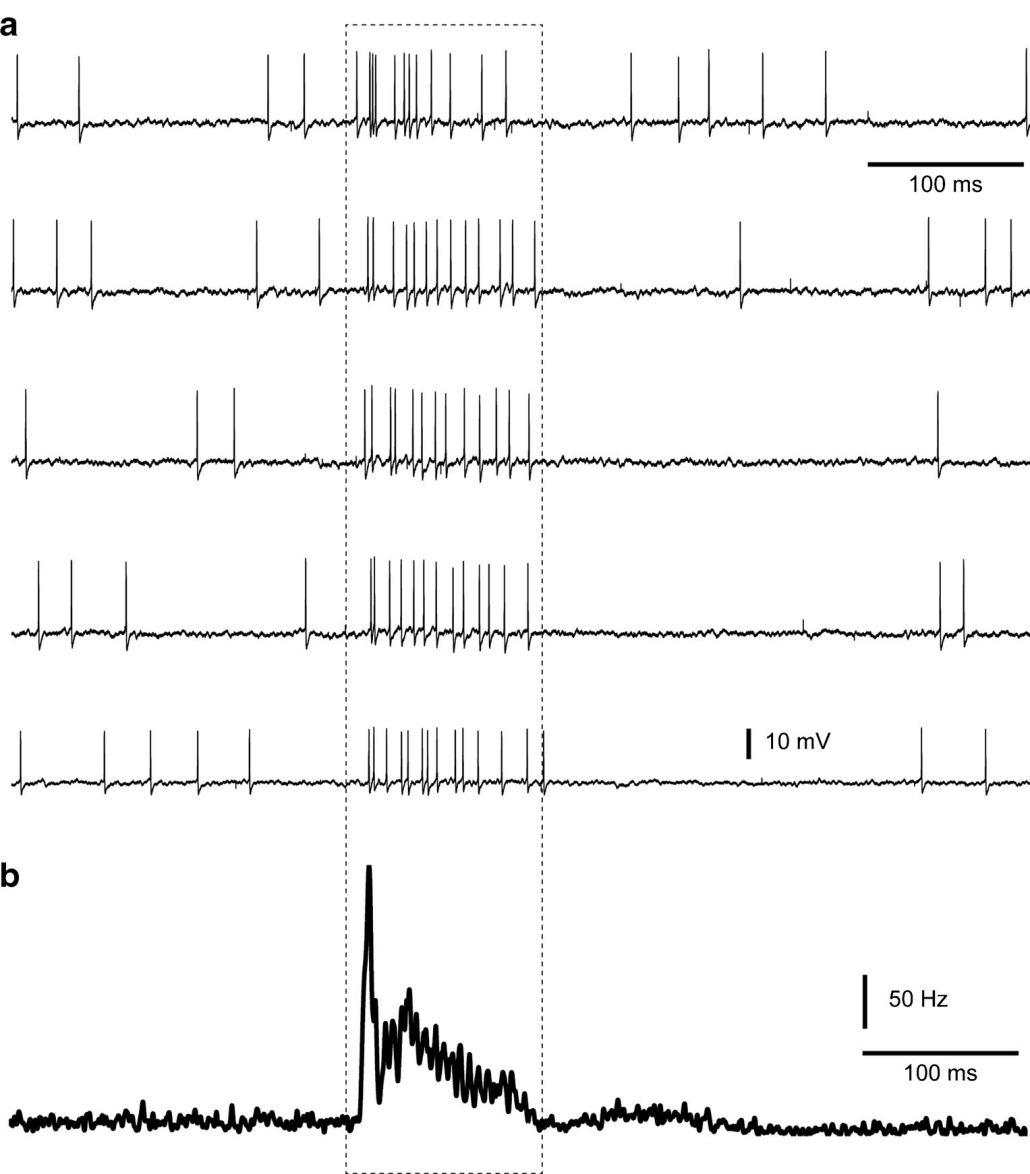
Stimulation protocol of combined direct SCT burst stimulation and skin burst stimulation. **a** Five raw traces to illustrate the intensity of the response. **b** For the same neuron, KDE plot of the full set of responses (*N* = 200 stimulations) in this type of protocol

Activation of SCTs can also be obtained using skin stimulation, as a proportion of the spinal neurons that projects through the SCTs are activated by skin afferents and the AIN neurons can be prominently excited by input from the skin [3] (Fig. 1). The dorsal column nuclei, which is the other source of cutaneous mf input to the intermediate cerebellum, does not provide synapses to the AIN [9] or at least provides such synapses much more rarely than spinocerebellar axons [28]. For the first type of protocol, we wanted to combine the SCT input with another input that could serve to depolarize the AIN neuron and in this way increase the activation of NMDA receptors [2]. We found that electrical stimulation of skin afferents in bursts provided a maintained excitation of these neurons for the duration of the burst. This type of burst input was combined with simultaneous burst activation of the SCT and the combination generated highly intense spike responses in the AIN neurons (Fig. 3).

**Fig. 4:**
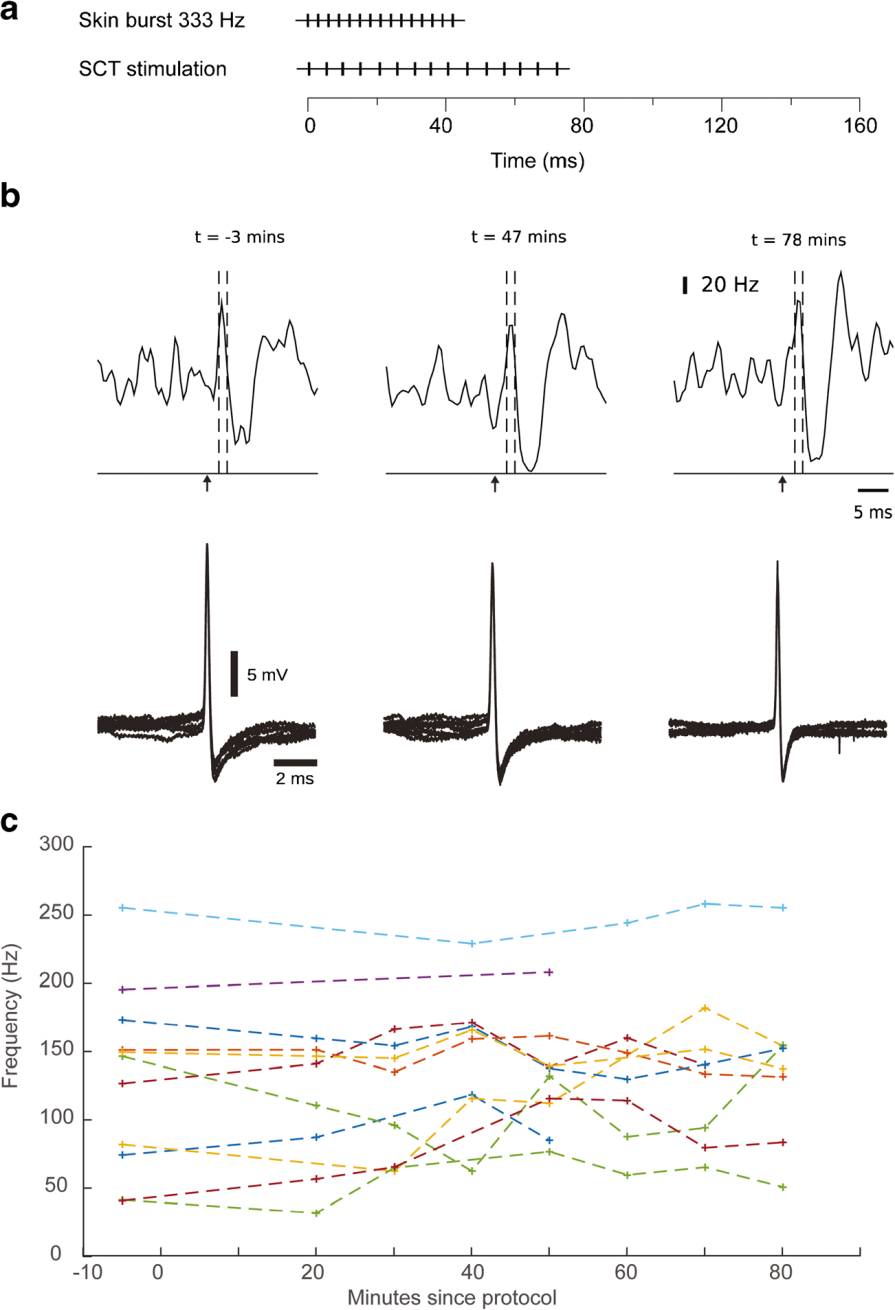
Responses evoked by single shock SCT stimulation before and after the stimulation protocol. **a** Stimulation protocol of combined direct SCT burst stimulation and skin burst stimulation. The intention of the protocol is to induce a plastic change in the response amplitude to single shock SCT stimulation in the recorded AIN neuron. **b** *Top*, KDE plots for the responses (*N* = 200) evoked at three selected time points. *Arrows* indicate the time point of stimulation. The *dashed lines* indicate the time period in which the response was quantified. *Bottom*, superimposed raw traces of the spikes (*N* = 10 per panel) recorded from at different time points. Note that the small drop in spiking activity in the KDE plot around the stimulation time point is due to that some spikes occurring in conjunction with the stimulus artifact was hard to identify. This had no effect on the analysis of the change in the response, as this was measured relative to a long prestimulus time window of activity. **c** Individual spiking frequencies during the response time window for all neurons

After the termination of the burst protocol (Fig. 4a), the responses evoked by a single shock SCT stimulation was monitored for up to 80 min and compared to the response to the same input recorded before the onset of the protocol (Fig. 4b). The first component of the response evoked by the SCT, which occurred before the onset of the first wave of inhibition (cf. [3]), was quantified typically for a time window of 1.5–4.0 ms after the onset of the SCT stimulation. SCT evoked responses in all *N* = 11 cells. For each time point (10, 20, 30, 40, 50, 60, 70, 80, and 90 min after stimulation), there was experimental data from seven to ten out of *N* = 11 neurons. As can be seen in Fig. 4c, the inter-cell variance in firing activity is relatively large, which is the rationale for using the Wilcoxon signed-rank test as instead of calculating a mean for all cells in each time bin. The result of the statistical analysis can be found in Table 1; the raw data is provided in Fig. 4a to make it possible for the reader to judge the probability that there were any substantial and reliable changes induced by the protocol across the population of neurons recorded.

**Table 1:** Number of cells with positive changes in response amplitude versus the total number of cells shown, respectively, for each stimulation protocol and each time point. If there is no systematic potentiation or depression, on average, there should be as many cells with positive as with negative changes in response amplitude

Time (min)	Number of cells with positive response change	Total number of cells	Wilcoxon signed-rank test
Combined SCT and skin burst protocol			
15–25	4	8	1
25–35	3	8	1
35–45	5	8	0.54
45–55	7	10	0.28
55–65	3	7	1
65–75	6	9	0.65
75–85	4	8	0.84
85–95	6	7	0.05
Skin burst and simultaneous single IO stimulation protocol			
5–15	2	6	0.84
15–25	3	5	0.63
25–35	3	5	0.43
35–45	3	5	0.63
Skin burst and delayed single IO stimulation protocol			
5–15	1	5	0.31
15–25	2	5	0.31
25–35	3	4	0.25

Wilcoxon signed-rank test gives the *P* value (*rightmost column*) for the null hypothesis that there is no potentiation. Note that in pure chance data, 1 out of the 20 *P* values is expected to fall within the 5% range, which is also the case here (1 out of the 15 comparisons made)

### Effect of the Skin Burst and Simultaneous, Single IO Stimulation Protocol

**Fig. 5:**
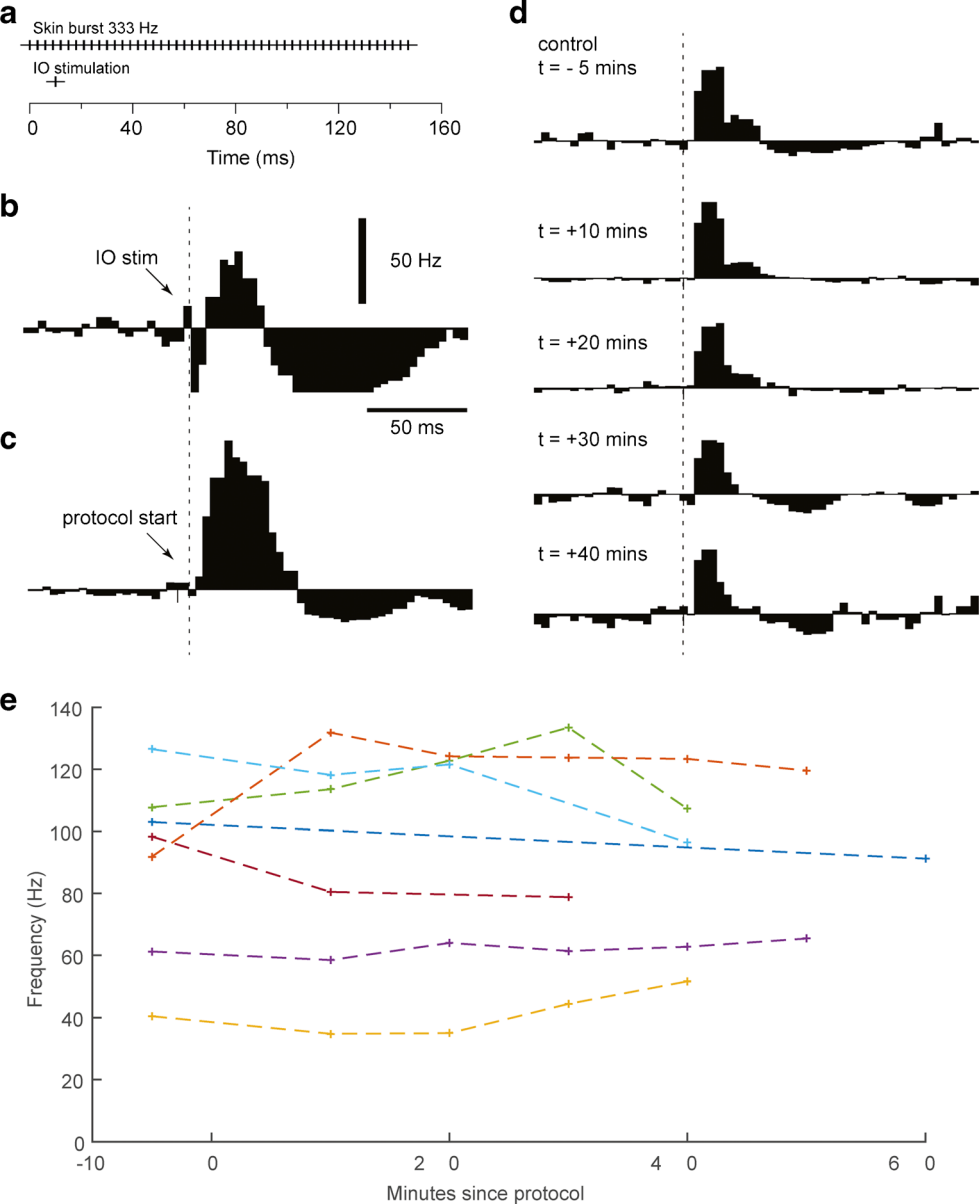
The effects of the simultaneous IO and skin burst activation protocol for a sample AIN neuron. **a** Stimulation protocol of simultaneous IO and skin burst activation. The intention of the protocol is to induce plastic changes in the response amplitudes to single-pulse electrical skin stimulation. **b** Net peristimulus histogram of responses evoked by IO stimulation (*N* = 200 responses, bin width 5 ms). **c** Net peristimulus histogram of responses evoked during the protocol (*N* = 200 repetitions), which consisted of a single IO stimulation and a burst stimulation to the skin applied at the same time point. **d** Development over time of the net response to single shock stimulation to the skin. Times are given relative to the start point of the stimulation protocol (for the control) and relative to the end point of the stimulation protocol (for all other histograms), respectively. Each histogram was obtained from 100–200 repetitions of the stimulation. **e** Spiking frequencies for each individual cell during the response time window

In the cerebellar cortex, simultaneous burst stimulation of the excitatory parallel fiber (PF) synapses and a single shock stimulation of the cfs by a stimulation electrode in the IO are highly effective to induce long-term potentiation of PF inputs in Purkinje cells and their afferent interneurons [14]. We applied a similar protocol to AIN neurons, but replaced the PF stimulation with cutaneous input using localized electrical skin stimulation to a skin site that provided effective at least some excitation but was located outside the cf receptive field of the cell and therefore produced submaximal excitatory input to the cell [3] (Fig. 5a). As previously described [4], we carefully limited our data set to those AIN neurons in which the IO stimulation at low stimulation intensities (≤50 μA) evoked a characteristic and distinct response sequence including early excitation-inhibition, representing the direct cf excitation of the DCN neuron followed by cf-driven, powerful, Purkinje cell inhibition and an ensuing postinhibitory rebound response (Fig. 5b, often also followed by inhibition after the rebound). Simultaneous IO stimulation and skin burst stimulation evoked substantial responses in the AIN neuron (Fig. 5c). However, the response to the single skin stimulation pulse, which was the test input, appeared to hardly change at all, neither in magnitude nor in temporal topography, after the protocol (Fig. 5d). These findings were repeated for up to six neurons and four time points (Fig. 5e), and in none of the cases could the null hypothesis that there was no post-protocol potentiation of the response be rejected (Table 1).

### Effect of the Skin Burst and Delayed Single IO Stimulation Protocol

**Fig. 6:**
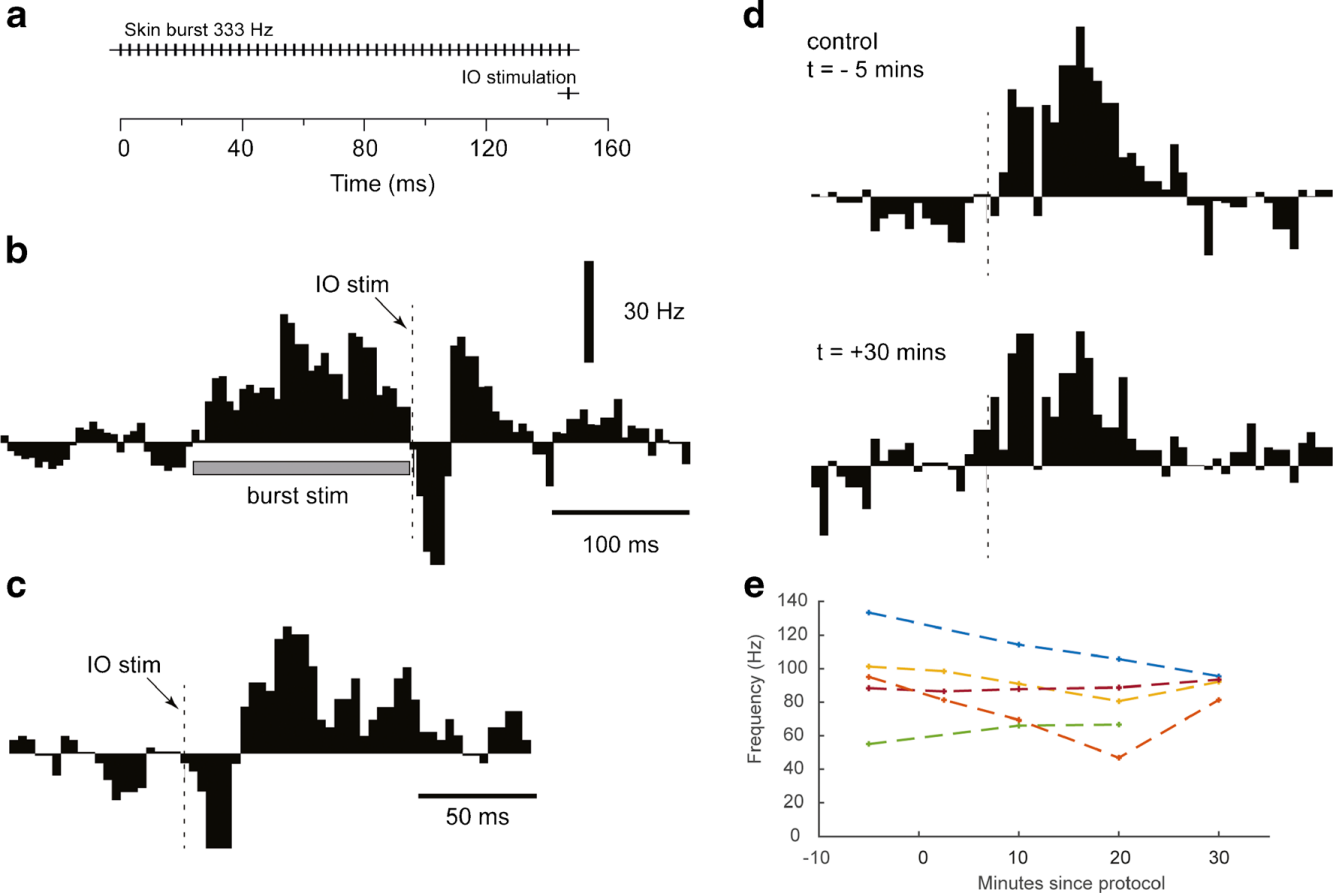
The effects of the skin burst and delayed single IO stimulation protocol for a sample AIN neuron. **a** Stimulation protocol of skin burst and delayed IO stimulation. The intention of the protocol is to induce plastic changes in the response amplitudes to single-pulse electrical skin stimulation. **b** Net peristimulus histogram of the spike responses to the protocol (*N* = 200 repetitions, bin width 5 ms). **c** Net peristimulus histogram of the spike responses to IO stimulation alone (*N* = 50 repetitions). Note the more expanded time base compared to **a**. **d** Net peristimulus histograms of the spike responses evoked to single-shock skin stimulation before and after the protocol (*N* = 100–200 repetitions). **e** Spiking frequencies for each individual cell during the response time window

Since some of the theoretical arguments for plasticity in the mf-DCN synapse come from the field of classical conditioning of the eyeblink reflex [32], we also tested a protocol which resembles that used for inducing classically conditioned responses. This protocol was similar to the preceding protocol in that it used a skin burst stimulation to obtain intense excitation of the AIN neuron, but the single-pulse IO stimulation was applied after the termination of the burst rather than at the time of its onset (delay conditioning protocol) (Fig. 6a, b). As described above, the AIN neurons were confirmed to have a prominent and characteristic response to the IO stimulation (Fig. 6c). As above, the responses obtained before and after the protocol were compared and were again found to be remarkably similar, both with respect to response magnitude and temporal topography (Fig. 6d). The summarized data, obtained from *N* = 5 AIN neurons (Fig. 5e), confirmed that no significant changes in response magnitude occurred (Table 1).

## Discussion

In the present study, we tested whether the efficacy of mf input to DCN neurons could be altered using any out of the three different types of stimulation protocols in the adult cerebellum in vivo. Essentially, no effects were obtained over the first 10–90 min following the termination of any of the stimulation protocols. This is in contrast to the dramatic effects obtained in the neurons of the cerebellar cortex over a similar time span using related stimulation protocols [11, 12, 14, 16]. We conclude that at least in terms of efficacy and speed of induction, plasticity in the mf-DCN neuron synapse appears to be much less effective than in the parallel fiber synapses in the cortex. The potential consequences for our understanding of the function of the cerebellum in learning and adaptation are discussed.

It could of course be argued that the data set was limited and that other results would have been obtained with more data. While this is always true for any data, visual inspection of the time course of net changes in post-protocol response amplitude in all cells revealed no trend in the data in either direction, again in contrast to data from the cerebellar cortex (see above). On basis of the absence of any trend, we could hence not defend extending the data acquisition in these very time consuming and difficult experiments. At the same time, the scientific community has repeatedly realized that it is important that also negative findings are published [17, 18], even though it can be argued that they are less conclusive.

First, it is important to point out that these findings do not imply that mf-interpositus plasticity does not exist in the adult cerebellum. It is of course possible that other protocols that we did not try would have been more effective. Perhaps a more likely possibility is that more long-term protocols and longer duration AIN cell recordings could have provided a different answer. In experiments of classically conditioned eyeblink responses, for example, effects in the Purkinje cell responses start to emerge at the same time scale as we were looking at here but they evolve substantially for hours after [11]. It should also be noted that we used much shorter intertrial intervals than in the latter paper, so comparisons cannot be made directly. The structural changes observed in mf collaterals to the DCN after repeated training protocols [5] was obtained only after several days of training, but it was not possible for us to follow single neurons for a comparable amount of time. In comparison with mf synaptic plasticity in juvenile slices [26, 27], however, the time scales were comparable but the results were very different. Notably, apart from age differences there was also a striking difference between the protocols: an important component in the slice work was the presence of a 20–25-mV hyperpolarization of the DCN neuron during the stimulation of the mf synapses, which lasted for at least 150 ms in order to initiate a postinhibitory rebound [26]. In the in vivo setting, such a hyperpolarization would appear to translate to a simultaneous activation of all of the afferent Purkinje cells to firing rates of 200–300 Hz for the duration of the period of inhibition [4], which appears to be a completely unlikely scenario in the adult cerebellum. This does not necessarily exclude that the fundamental plasticity mechanism described in the slice [26, 27] applies in vivo, but may suggest that the mechanism could operate on a much slower time course in the adult cerebellum in vivo, where these extreme cases of concerted Purkinje cell activity may not appear.

An interesting aspect is the contrast to the effects observed in cerebellar cortical neurons using similar protocols and, in at least one case, comparable recording times [14, 16]. This suggests that there is likely to be a difference between the interpositus cells and the cortical neurons at least in their propensity for plasticity of excitatory inputs. From a functional point of view, this may make sense. The limb areas of the AIN are an integral part of a motor command loop, which via direct connections to rubrospinal and thalamocortical neurons innervating the motor cortex can strongly influence the activity of spinal premotor interneurons [3]. These interneurons are probably very important for the synergy selection, i.e., which muscles are to be activated at what time during the execution of a complex, well-trained movement [30]. Since some of these interneurons provide feedback to the cerebellum, directly or via the lateral reticular nucleus, and since this feedback is the information that is provided by the spinocerebellar mf-DCN synapses, this synaptic linkage can be important for associating and linking specific synergy patterns into compound movements. As they are one of the fundaments of the core motor command loop, it may be important to let them become stabilized after development when basic movement patterns/synergy patterns have been acquired. Fine-tuning of the drive of these synergy patterns during specific phases/contexts of a movement can be achieved via the cerebellar cortex and its inhibitory control of the AIN neurons. This fine-tuning can be minor adaptations required by changes in muscle strength over time or context-dependent factors, for example, which do not require a change in the fundamental movement patterns. Such adaptations must be allowed to occur more rapidly and could be primarily brought about by alterations in the cortical network—this would be an explanation for the different propensities for input plasticity in the cortex as compared to the AIN neurons.

## References

[1] Aizenman CD, Linden DJ (2000). Rapid, synaptically driven increases in the intrinsic excitability of cerebellar deep nuclear neurons. Nat Neurosci.

[2] Anchisi D, Scelfo B, Tempia F (2001). Postsynaptic currents in deep cerebellar nuclei. J Neurophysiol.

[3] Bengtsson F, Jorntell H (2014). Specific relationship between excitatory inputs and climbing fiber receptive fields in deep cerebellar nuclear neurons. PLoS One.

[4] Bengtsson F, Ekerot CF, Jorntell H (2011). In vivo analysis of inhibitory synaptic inputs and rebounds in deep cerebellar nuclear neurons. PLoS One.

[5] Boele HJ, Koekkoek SK, de Zeeuw CL, Ruigrok TJ (2013). Axonal sprouting and formation of terminals in the adult cerebellum during associative motor learning. J Neurosci.

[6] Ekerot CF, Jorntell H, Garwicz M (1995). Functional relation between corticonuclear input and movements evoked on microstimulation in cerebellar nucleus interpositus anterior in the cat. Exp Brain Res.

[7] Freeman JH, Steinmetz AB (2011). Neural circuitry and plasticity mechanisms underlying delay eyeblink conditioning. Learn Mem.

[8] Garwicz M, Ekerot CF (1994). Topographical organization of the cerebellar cortical projection to nucleus interpositus anterior in the cat. J Physiol.

[9] Gerrits NM, Voogd J, Nas WS (1985). Cerebellar and olivary projections of the external and rostral internal cuneate nuclei in the cat. Exp Brain Res.

[10] Hoebeek FE, Witter L, Ruigrok TJ, de Zeeuw CI (2010). Differential olivo-cerebellar cortical control of rebound activity in the cerebellar nuclei. Proc Natl Acad Sci U S A.

[11] Jirenhed DA, Bengtsson F, Hesslow G (2007). Acquisition, extinction, and reacquisition of a cerebellar cortical memory trace. J Neurosci.

[12] Jirenhed DA, Bengtsson F, Jorntell H (2013). Parallel fiber and climbing fiber responses in rat cerebellar cortical neurons in vivo. Front Syst Neurosci.

[13] Jorntell H, Ekerot CF (1999). Topographical organization of projections to cat motor cortex from nucleus interpositus anterior and forelimb skin. J Physiol.

[14] Jorntell H, Ekerot CF (2002). Reciprocal bidirectional plasticity of parallel fiber receptive fields in cerebellar purkinje cells and their afferent interneurons. Neuron.

[15] Jorntell H, Ekerot CF (2003). Receptive field plasticity profoundly alters the cutaneous parallel fiber synaptic input to cerebellar interneurons in vivo. J Neurosci.

[16] Jorntell H, Ekerot CF (2011). Receptive field remodeling induced by skin stimulation in cerebellar neurons in vivo. Front Neural Circuits.

[17] Knight J (2003). Negative results: null and void. Nature.

[18] Matosin N, Frank E, Engel M, Lum JS, Newell KA (2014). Negativity towards negative results: a discussion of the disconnect between scientific worth and scientific culture. Dis Model Mech.

[19] Matsushita M (1999). Projections from the lowest lumbar and sacral-caudal segments to the cerebellar nuclei in the rat, studied by anterograde axonal tracing. J Comp Neurol.

[20] Matsushita M (1999). Projections from the upper lumbar cord to the cerebellar nuclei in the rat, studied by anterograde axonal tracing. J Comp Neurol.

[21] Matsushita M, Gao X (1997). Projections from the thoracic cord to the cerebellar nuclei in the rat, studied by anterograde axonal tracing. J Comp Neurol.

[22] Matsushita M, Xiong G (1997). Projections from the cervical enlargement to the cerebellar nuclei in the rat, studied by anterograde axonal tracing. J Comp Neurol.

[23] Matsushita M, Yaginuma H (1995). Projections from the central cervical nucleus to the cerebellar nuclei in the rat, studied by anterograde axonal tracing. J Comp Neurol.

[24] Ohyama T, Nores WL, Medina JF, Riusech FA, Mauk MD (2006). Learning-induced plasticity in deep cerebellar nucleus. J Neurosci.

[25] Porrill J, Dean P (2007). Cerebellar motor learning: when is cortical plasticity not enough?. PLoS Comput Biol.

[26] Pugh JR, Raman IM (2006). Potentiation of mossy fiber EPSCs in the cerebellar nuclei by NMDA receptor activation followed by postinhibitory rebound current. Neuron.

[27] Pugh JR, Raman IM (2008). Mechanisms of potentiation of mossy fiber EPSCs in the cerebellar nuclei by coincident synaptic excitation and inhibition. J Neurosci.

[28] Quy PN, Fujita H, Sakamoto Y, Na J, Sugihara I (2011). Projection patterns of single mossy fiber axons originating from the dorsal column nuclei mapped on the aldolase c compartments in the rat cerebellar cortex. J Comp Neurol.

[29] Raymond JL, Lisberger SG (1998). Neural learning rules for the vestibulo-ocular reflex. J Neurosci.

[30] Santello M, Baud-bovy G, Jorntell H (2013). Neural bases of hand synergies. Front Comput Neurosci.

[31] Spanne A, Jorntell H (2013). Processing of multi-dimensional sensorimotor information in the spinal and cerebellar neuronal circuitry: a new hypothesis. PLoS Comput Biol.

[32] Weeks AC, Connor S, Hinchcliff R, Leboutillier JC, Thompson RF, Petit TL (2007). Eye-blink conditioning is associated with changes in synaptic ultrastructure in the rabbit interpositus nuclei. Learn Mem.

[33] Wu HS, Sugihara I, Shinoda Y (1999). Projection patterns of single mossy fibers originating from the lateral reticular nucleus in the rat cerebellar cortex and nuclei. J Comp Neurol.

[34] Wulff P, Schonewille M, Renzi M, Viltono L, Sassoe-pognetto M, Badura A, Gao Z, Hoebeek FE, van Dorp S, Wisden W, Farrant M, de Zeeuw CI (2009). Synaptic inhibition of Purkinje cells mediates consolidation of vestibulo-cerebellar motor learning. Nat Neurosci.

